# Distinguishing Hepatocellular Carcinoma from Cirrhotic Regenerative Nodules Using MR Cytometry

**DOI:** 10.3390/cancers17071204

**Published:** 2025-04-01

**Authors:** Xiaoyu Jiang, Mary Kay Washington, Manhal J. Izzy, Ming Lu, Xinqiang Yan, Zhongliang Zu, John C. Gore, Junzhong Xu

**Affiliations:** 1 Vanderbilt University Institute of Imaging Science, Vanderbilt University Medical Center, Nashville, TN 37232, USA; ming.lu@vumc.org (M.L.); xinqiang.yan@vumc.org (X.Y.); zhongliang.zu@vumc.org (Z.Z.); john.gore@vumc.org (J.C.G.); junzhong.xu@vumc.org (J.X.); 2 Department of Radiology and Radiological Sciences, Vanderbilt University Medical Center, Nashville, TN 37232, USA; 3 Department of Pathology, Microbiology and Immunology, Vanderbilt University, Nashville, TN 37232, USA; kay.washington@vumc.org; 4 Department of Medicine, Vanderbilt University Medical Center, Nashville, TN 37232, USA; manhal.izzy@vumc.org; 5 Department of Biomedical Engineering, Vanderbilt University, Nashville, TN 37232, USA; 6 Department of Physics and Astronomy, Vanderbilt University, Nashville, TN 37232, USA

**Keywords:** cell size, cellularity, MR cytometry, diffusion, hepatocellular carcinoma, cirrhotic regenerative nodule

## Abstract

Background and Objectives: Current guidelines recommend contrast-enhanced CT/MRI as confirmatory imaging tests for diagnosing hepatocellular carcinoma (HCC). However, these modalities are not always able to differentiate HCC from benign/dysplastic nodules that are commonly observed in cirrhotic livers. Consequently, many lesions require either pathological confirmation via invasive biopsy or surveillance imaging after 3–6 months, which results in delayed diagnosis and treatment. We aimed to develop noninvasive imaging biomarkers of liver cell size and cellularity, using magnetic resonance imaging (MRI), and to assess their utility in identifying HCC. Methods: MR cytometry combines measurements of water diffusion rates over different times corresponding to probing cellular microstructure at different spatial scales. Maps of microstructural properties, such as cell size and cellularity, are derived by fitting voxel values in multiple diffusion-weighted images to a three-compartment (blood, intra-, and extracellular water) model of the MRI signal. This method was validated in two phases: (1) histology-driven simulations, utilizing segmented histological images of different liver pathologies, and (2) ex vivo MR cytometry performed on fixed human liver specimens. Results: Both simulations and ex vivo MR cytometry of fixed human liver specimens demonstrated that HCC exhibits significantly smaller cell sizes and higher cellularities compared to normal liver and cirrhotic regenerative nodules. Conclusion: This study highlights the potential of MR cytometry to differentiate HCC from non-HCC lesions by quantifying cell size and cellularity in liver tissues. Our findings provide a strong foundation for further research into the role of MR cytometry in the noninvasive early diagnosis of HCC.

## Simple Summary

Current guidelines rely on contrast-enhanced CT/MRI for hepatocellular carcinoma (HCC) diagnosis, but these often fail to distinguish HCC from benign/dysplastic nodules in cirrhosis, leading to invasive biopsies or delayed treatment. We developed MR cytometry, a noninvasive diffusion-MRI-based method quantifying liver cell size and cellularity by modeling water diffusion across different diffusion times. The technique was validated through (1) histology-driven simulations and (2) ex vivo imaging of fixed human liver specimens. Results showed that HCC exhibits smaller cell sizes and higher cellularity compared to normal liver and regenerative nodules. These microstructural biomarkers could improve early HCC detection without invasive procedures. MR cytometry demonstrates promise as a diagnostic tool, warranting further clinical investigation to enhance HCC differentiation and management.

## 1. Introduction

Early diagnosis of hepatocellular carcinoma (HCC)—currently the fifth most common cancer and the third leading cause of cancer-related deaths worldwide—can substantially improve the 5-year survival rate [1,2]. In the United States, 90% of HCC cases develop in patients with preexisting cirrhosis [3,4], making it critical to differentiate between HCC and cirrhotic regenerative nodules (CRNs) [5], both of which commonly appear in cirrhotic livers. While contrast-enhanced CT and MRI, guided by the Liver Imaging Reporting and Data System (LI-RADS), are the standard imaging tools recommended by clinical guidelines, they sometimes fail to distinguish CRNs from HCC [6]. Both CRNs and HCC may exhibit similar enhancement patterns on imaging, such as rapid washout, making it occasionally challenging to definitively distinguish between them based on imaging appearance alone. As a result, many lesions, classified as LR-4 or LR-M, require biopsy—the current, albeit imperfect, diagnostic gold standard—or follow-up imaging every 3 to 6 months in the case of LR3, potentially delaying diagnosis and treatment.

A key cytological difference between early-stage HCC, which is often well-differentiated, and CRNs lies in their cellular characteristics [7]. Early-stage HCC typically constitutes cells smaller than normal hepatocytes and has a cell density approximately twice that of normal liver tissue, whereas CRNs resemble normal hepatocytes [7,8]. Additionally, well-differentiated HCC is characterized by larger, more prominent nucleoli and an increased nucleus-to-cytoplasm (N/C) ratio compared to CRNs [9]. Currently, this information can only be obtained through liver biopsy.

Significant efforts have been made to develop noninvasive imaging methods that characterize these cellular characteristics. Diffusion MRI (dMRI) is a promising technique that examines the restriction and hindrance of water molecules diffusing in biological tissues, providing unique insight into tissue microstructure. The widely used apparent diffusion coefficient (ADC), derived from dMRI, has been shown to help differentiate HCC from surrounding liver tissue and potentially predict its aggressiveness or differentiation grade, without the need for contrast injection [10,11,12]. The underlying mechanism is that higher cellularity within a tumor leads to restricted water diffusion and a lower ADC value, indicating a more poorly differentiated HCC [13]. However, ADC values reflect averaged diffusion properties across all structures and length scales, yielding ambiguous microstructural information. The correlation between ADC values and histopathological findings may not always be strong and can vary depending on the study [10,11].

Recently, a class of advanced dMRI methods has been developed to tackle this limitation. Specifically, these methods use multi-compartment biophysical models and acquisitions with multi-b and multi-diffusion times to estimate mean cell size and cellularity, such as VERDICT (vascular, extracellular, and restricted diffusion for cytometry in tumors) [14], IMPULSED (imaging microstructural parameters using limited spectrally edited diffusion) [15,16,17,18], JOINT (IMPULSED combined with transcytolemmal water exchange) [18], and POMACE (pulsed and oscillating gradient MRI for assessment of cell size and extracellular space) [19]. Because these methods non-invasively characterize microstructural information at the cellular level, we have termed this class of techniques MR cytometry. To date, MR cytometry is almost exclusively utilized for cancer imaging [20]. Previous research has demonstrated the feasibility of MR cytometry in patients with prostate [21], breast cancer [16,22,23], and ovarian cancer [24], employing a clinically feasible protocol known as IMPULSED on 3T clinical scanners [15,16,21,22,23,24,25]. The accuracy of MRI-derived cell size and cellularity has been rigorously validated through in silico simulations [26], in vitro experiments [16,26], and in vivo animal models [20]. Recently, we achieved a significant milestone by being the first to adapt and implement MR cytometry for in vivo imaging of healthy human and rodent livers [15], establishing the feasibility of measuring cell size and cellularity within hepatic tissues.

Building on this foundation, we hypothesize that MR cytometry can quantify differences in cell size and cellularity between early-stage HCC and CRNs. In this pilot study, we tested this hypothesis by conducting histology-based simulations and performing ex vivo MR cytometry and histology analysis on fixed human specimens.

## 2. Materials and Methods

### 2.1. Theory of MRI Cytometry

Within the framework of Temporal Diffusion Spectroscopy (TDS), MR cytometry [20] was initially developed for quantifying cell sizes in solid tumors. It derives microstructural parameters including mean cell size d, intracellular volume fraction $v_{in}$, and intra/extra cellular diffusion coefficients $D_{in}$/$D_{ex}$ from a limited number of diffusion measurements with varying clinically achievable diffusion times (i.e., from 5 to 70 ms). Strictly, d measures the separation of boundaries that restrict free diffusion, but in normal tissues and several other conditions the cell membrane is the major cause of restrictions. The key to applying MR cytometry is determining whether the size of the compartment of interest can be sensitized by water diffusion within the given diffusion times. The range of sizes of most interest in liver tissues is from 5 um to 25 µm (e.g., hepatocytes approximately 15–25 µm [15,27], cancer cells approximately 10–15 µm [28]). These correspond to diffusion times on the order of 5–70 ms, which can be achieved using a combination of OGSE (oscillating gradient spin echo) and PGSE (pulsed gradient spin echo) measurements on clinical scanners. Therefore, it is possible to assess liver microstructure by combining measurements of water diffusion rates over different time scales.

The normalized, fat-saturated, diffusion-weighted signals are expressed as the sum of restricted diffusion in liver cells, hindered diffusion in the extracellular extravascular space, and blood flow dephasing, namely,

(1) $$S_{total}\left(b,t_{diff}\right)=\left(1-f_{IVIM}\right)\times \left(v_{in}S_{in}+\left(1-v_{in}\right)S_{ex}\right)+f_{IVIM}\times S_{blood}$$

$$where\;S_{in}\left(b,t_{diff}\right)=\exp\left[-b\times {ADC}_{sphere}\right],\;S_{ex}\left(b,t_{diff}\right)=exp(-b\times D_{ex})$$

$$S_{blood}\left(b,t_{diff}\right)=exp(-b\times D^{*})$$

where *b* is the diffusion weighting factor, $t_{diff}$ is the effective diffusion time (≈1/4f for oscillating gradients with oscillation frequency f), $f_{IVIM}$ is the blood volume fraction from perfusion, and $v_{in}$ is the water volume fraction of intracellular extravascular space. ${ADC}_{sphere}$ is the apparent diffusion rate in an impermeable sphere with a diameter of d. $D_{ex}$ is the extracellular diffusion rate. $D^{*}$ reflects dephasing due to blood perfusion in capillaries and depends on factors such as the blood self-diffusion coefficient, blood flow velocity, and diffusion encoding waveforms. $S_{in}$, $S_{ex}$, and $S_{blood}$ are the diffusion MRI signal magnitudes per volume from the intracellular, extracellular extravascular, and intravascular spaces, respectively. Analytical expressions of $S_{in}$ and $S_{ex}$ acquired by cosine-modulated OGSE and PGSE sequences can be found in our previous publications [20].

In addition, due to increased cell membrane permeability in liver diseases [29], the diffusion-time-dependent influences of water exchange between intra- and extracellular compartments [18] have been taken into consideration. The effects of water exchange are ignored when $t_{diff}$ is short (e.g., 5 ms using OGSE) but are included in the diffusion signal model when $t_{diff}$ is long (e.g., ≥30 ms using either PGSE or STEAM) using the modified Kӓrger model [30]. This adapted signal model has been shown to improve the accuracy of MRI-derived cell size d, $v_{in}$, and cellularity in solid tissues. Details of the signal model have been published previously [18].

### 2.2. Validation Using Histology-Based Simulations

This study utilizes a Finite Difference (FD) method [31] to simulate diffusion signals for compartmental tissue structures derived from segmented histological images using MATI, an in-house diffusion MRI simulation toolbox. The tissue structures were segmented into intra- and extracellular spaces, characterized by restricted and hindered water diffusion, respectively. Diffusivities for intra- and extracellular spaces are 1.53 and 2 μm^2^/ms, respectively. The proton density of intra- and extracellular spaces was set to homogeneous. Homogeneous T2 relaxation times were set for all three compartments for simplicity. Permeabilities varied from 0 to 0.05 µm/ms with an interval of 0.01 µm/ms. The PGSE and OGSE acquisition parameters are the same as those in the in vivo MR cytometry protocol described in Section 2.4. For each pathology, six segmented histological images (2000 × 2000 pixels with a resolution of 0.5 × 0.5 µm^2^ per pixel) were used to generate six sets of diffusion data. Rician noise at three SNR levels (10, 20, and 50) were added to the simulated diffusion signals. MR cytometry analysis was conducted afterwards.

### 2.3. Validation Using Ex Vivo MRI Cytometry and Histology Analysis

**Figure 1:**
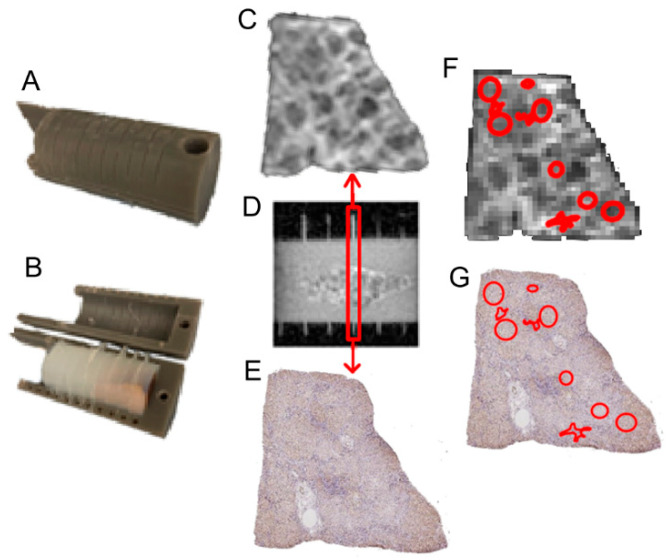
(**A**,**B**). 3D-printed sample holder; (**C**). Axial MR image acquired at a 4% agarose-filled slot in the sample holder, highlighted by a red rectangle; (**D**) Sagittal view of the sample holder with the specimen embedded in the 4% agarose; (**E**) Corresponding β-catenin stained histological image at the slot of the sample holder, highlighted by a red rectangle; (**F**,**G**) Regions of interest (ROIs, red shapes) on the histological image and MR image following coregistration.

Eleven human liver tissue samples with weights ranging from 1 to 15 g were obtained from the Western Division of the Cooperative Human Tissue Network at Vanderbilt University Medical Center. All the samples were immersed in 10% neutral buffered formalin for one week and then transferred to PBS solution for 24 h before MR imaging. The samples included normal liver samples (*n* = 3), samples with cirrhosis (*n* = 5), and HCC samples (*n* = 3). Each sample was embedded in a 3D-printed holder (Figure 1A,B) with 4% agarose for MRI measurements. This specially designed sample holder has evenly spaced slots with a slot width of 0.5 mm (the width of a razor blade) and 5 mm between slots. The 4% agarose-filled slots can be easily identified on MR images, which serve as landmarks for accurate slice coregistration between histology and MRI-derived parametric maps (Figure 1C–E). β-catenin (a cell membrane marker) staining was performed to visualize cell boundaries. Cell sizes and cellularities (number of nuclei cross-sections per mm^2^) were assessed by segmenting these β-catenin-stained pictures.

#### 2.3.1. Ex Vivo Imaging Protocol

MRI acquisitions were performed using a Varian/Agilent 4.7T scanner (Palo Alto, CA, USA). For PGSE experiments, diffusion gradient duration/separation were δ/Δ = 3/11 and 3/31 ms. OGSE acquisitions used a frequency of 50 Hz with δ/Δ = 20/25 ms. For STEAM experiments, diffusion gradient duration/separation were δ/Δ = 3/71 ms. Five b-values spaced at equal logarithmic intervals from 0 to either 1000 s/mm^2^ or the allowed maximum b value (limited by the maximum gradient strength of 360 mT/m in a single direction) were used to obtain estimates of the diffusion coefficient at each diffusion time. Other imaging parameters were as follows: slice thickness = 1 mm, slice numbers either 3 or 5 depending on the size of samples, in-plane resolution = 0.5 × 0.5 mm, TR = 2 s, and TE = 55 ms.

#### 2.3.2. Co-Registration Between Histology and Ex Vivo MRI

A 3-step approach was performed to co-register histology and ex vivo MR images.

Step 1. Two continuous 5 µm sections were obtained from the surface of each paraffin-embedded tissue block and were stained with H&E and β-catenin, respectively. Note that our customized tissue holder allows all the MR imaging slices to be collected from the surface plane of each paraffin-embedded tissue block.

Step 2. All the stained slides were scanned using an Aperio Versa 200 slide scanner (Leica Biosystems, Vista, CA, USA) at 20× to generate high-resolution digital images (0.5 × 0.5 µm^2^ per pixel). A total of 55 regions of interest (ROIs) representing different liver pathologies, including normal liver (*n* = 16), CRN (*n* = 28), and HCC (*n* = 11) samples, were drawn on the β-catenin-stained pictures by a liver pathologist (M.K.W). The high-resolution histological images were divided into small sub-images of size 1000 × 1000 pixels. Cellular parameters, such as average cell sizes and cellularities, were recorded using in-house algorithms written in MATLAB 2024b.

Step 3. Histological images were registered to MR images using a combination of Iterative Closest Point (ICP)-based rigid transformation included in the MATLAB Computer Vision Toolbox and 2D shape nonrigid registration (MATLAB codes available from http://fr.mathworks.com/matlabcentral/profile/authors/3793616-mohammad-rouhani, access on 10 December 2024), which captures both global and local alignment and deformation. ROIs drawn on the histological sections were then transformed to MRI-derived parametric maps using T2W MR images as bases (Figure 1F,G). For each ROI, cellular properties (e.g., cell size and cellularity) derived from histology and MR cytometry were summarized and compared.

### 2.4. MRI Cytometry Analysis

All diffusion data underwent four steps, including preprocessing (including registration and denoising), model selection, removal of blood perfusion, and derivation of microstructural maps. For the last step, the constraints for fitting parameters were based on physiologically relevant values [20,32]: 0 ≤ d ≤ 30 µm, 0 ≤ $v_{in}$ ≤ 1, 0 ≤ $D_{in}$ ≤ 3.0 µm^2^/ms, 0 ≤ ${ADC}_{ex}$ ≤ 3.0 µm^2^/ms, and 0 ≤ $\tau_{in}$ ≤ 1000 ms. Initial values for each fitting parameter were randomly selected from their ranges. All the data processing was performed using a MATLAB-based in-house software package that is available online (https://github.com/jzxu0622/mati, access on 10 December 2024). However, we focused only on MRI-derived d and $v_{in}$ in this study, with detailed explanations provided in the discussion.

For the ex vivo comparisons with histology, cellularity (the number of cell cross-sections per area) was calculated as $2\times {\left(\frac{3v_{in}}{2\pi }\right)}^{\frac{2}{3}}/d^{2}$ by assuming that solid tumors consist of spherical cancer cells densely packed on a face-centered cube grid.

### 2.5. Statistical Analysis

Group differences in (i) MRI-derived parameters among different types of liver tissues at different SNR levels and (ii) histology and MRI-derived parameters among different types of liver tissues were summarized using means and standard errors of the means and compared using one-way analysis of variance (ANOVA) with Bonferroni correction. The correlations between histology and MRI-derived cell sizes, and cellularities, were assessed using Spearman’s tau correlation coefficient.

## 3. Results

### 3.1. Histology-Based Simulations Confirmed Pathological Variations in Cell Size and Cellularity

**Figure 2:**
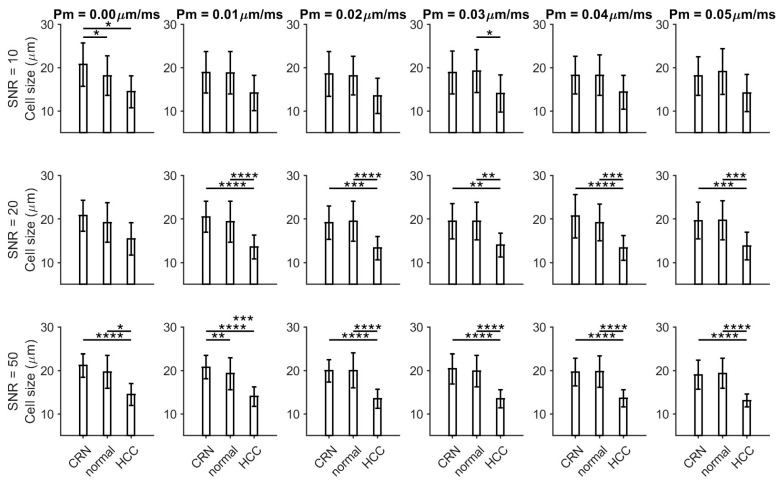
Fitted cell sizes from histology-based simulated diffusion signals for normal liver tissues, cirrhotic regenerative nodules (CRNs), and HCC at three different SNR levels (10, 20, and 50) and varying permeabilities. * *p* < 0.05, ** *p* < 0.01, *** *p* < 0.001, and **** *p* < 0.0001 as measured by one-way analysis of variance (ANOVA) with Bonferroni correction.

**Figure 3:**
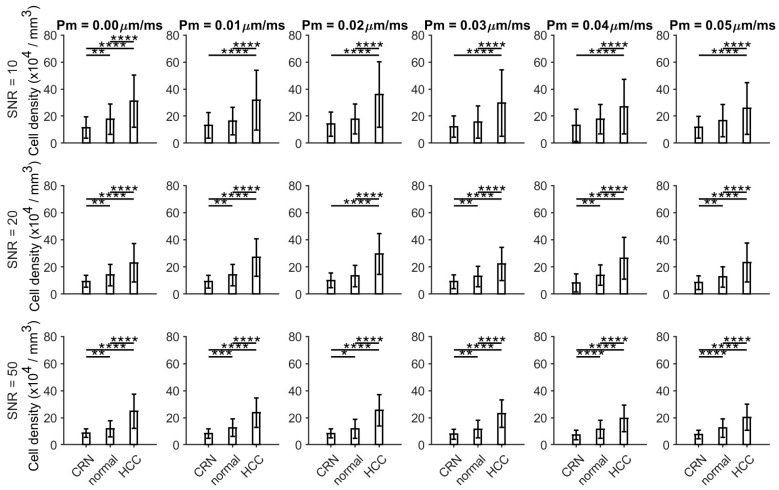
Fitted cell densities from histology-based simulated diffusion signals for normal liver tissues, cirrhotic regenerative nodules (CRNs), and HCC at three different SNR levels (10, 20, and 50) and varying permeabilities. * *p* < 0.05, ** *p* < 0.01, *** *p* < 0.001, and **** *p* < 0.0001 as measured by one-way analysis of variance (ANOVA) with Bonferroni correction.

As shown in Figure 2 and Figure 3, the histology-based simulations demonstrate that HCC exhibits smaller fitted cell sizes (~15 µm) and higher fitted cellularities (~80 × 10^4^/mm^2^) compared to CRNs and normal conditions across all SNR levels and varying permeabilities, with these differences becoming more pronounced at higher SNRs. CRN and normal liver tissues show relatively similar fitted cell sizes, while CRN tissue displays significantly lower cellularities than normal liver tissues at SNR values of 20 and 50. The simulations suggest that an SNR of 20 is sufficient to differentiate between HCC and other conditions.

### 3.2. Ex Vivo MR Cytometry Characterized Different Pathological Conditions

**Figure 4:**
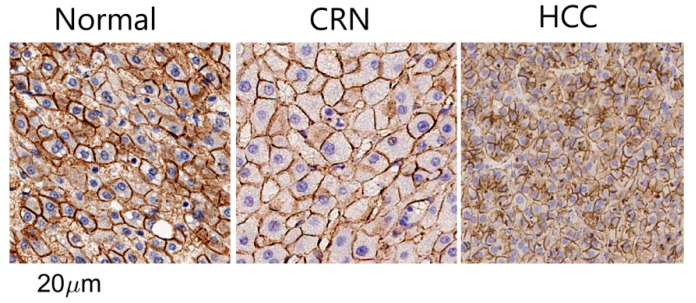
Representative β-catenin stained histological pictures for normal liver tissues, cirrhotic regenerative nodules (CRNs), and HCC.

**Figure 5:**
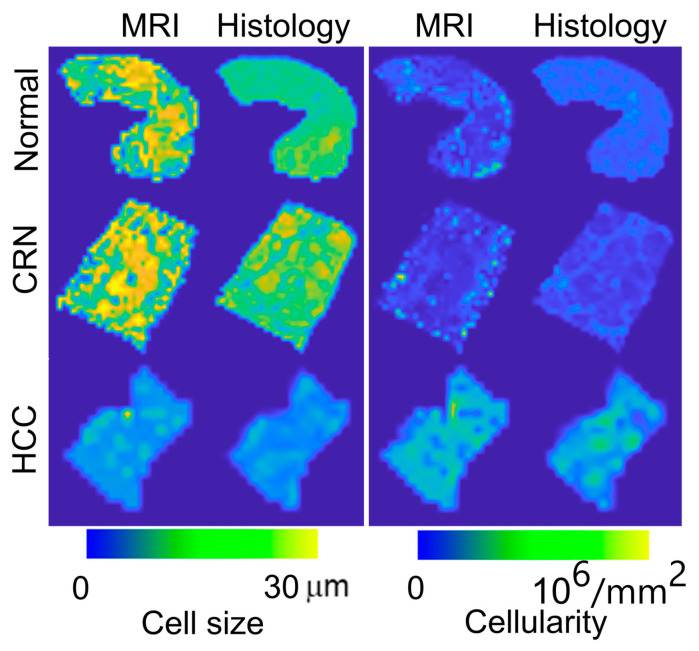
β-catenin-stained histological pictures and corresponding MRI and histology-derived maps of cell size and cellularity from three human liver specimens, including a normal liver sample, a sample with cirrhosis, and an HCC sample.

Figure 4 shows representative β-catenin-stained histologic sections, while Figure 5 presents MRI- and histology-derived maps of cell size and cellularity for three human liver samples, including a normal liver sample, an HCC sample, and a sample with cirrhosis. Strong membranous β-catenin staining revealed significant morphological differences among the different liver pathological conditions. Most cells in normal liver and CRN tissues are hepatocytes, which are bigger than tumor cells in HCC. Both MRI and histology-derived maps of cell size and cellularity clearly indicate that HCC has smaller cell sizes and higher cellularities compared to normal liver tissue and CRN tissue.

**Figure 6:**
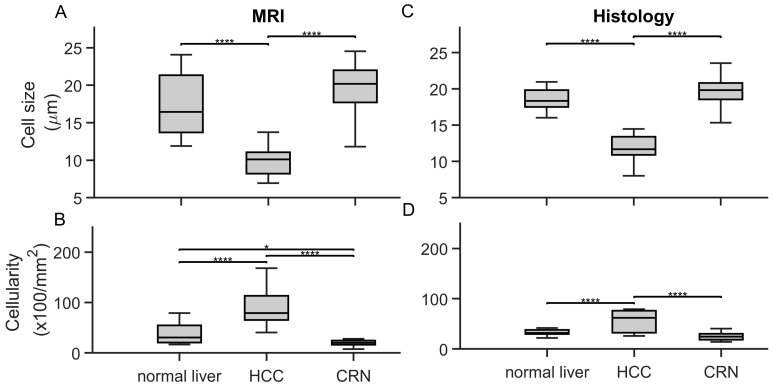
Box-and-whisker plots of MRI-derived cell sizes (**A**) and cellularities (**B**) and histology-derived cell sizes (**C**) and cellularities (**D**) for liver ROIs with different pathologies. For all the box-and-whisker plots, the 25th-75th percentiles are blocked by the box, the red bands inside the box are the mean values, and the whiskers mark the SD. * *p* < 0.05 and **** *p* < 0.0001 as measured by one-way analysis of variance (ANOVA) with Bonferroni correction.

The average cell sizes and cellularities of different liver pathologies and conditions were measured by histology and MR cytometry and summarized using box-and-whisker plots (Figure 6). Histology-derived cell sizes for HCC are significantly smaller than those for normal liver and CRN tissues, while cellularities in HCC are significantly higher compared to both normal liver and CRN tissues, confirming that both cell size and cellularity could serve as biomarkers for detecting HCC. MRI-derived cell sizes and cellularities for different liver pathologies show similar trends, with HCC exhibiting significantly smaller cell sizes and higher cellularities compared to normal liver and CRN tissues.

**Figure 7:**
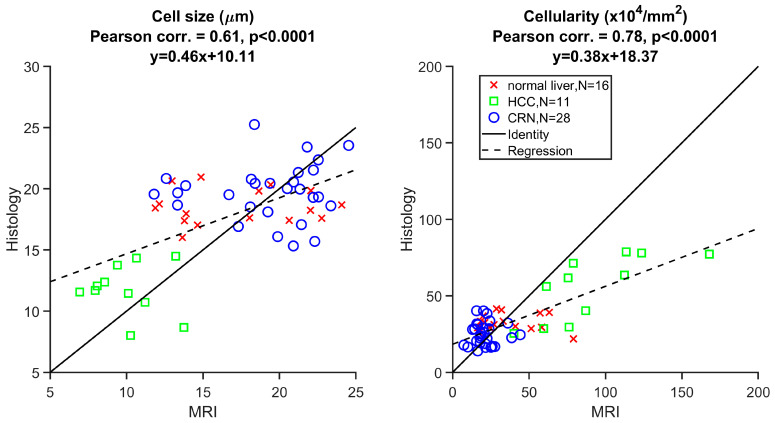
Correlation between histology and MRI-derived cellular parameters for all the liver ROIs. The solid and dashed lines represent the identity and regression, respectively.

Figure 7 demonstrates the linear correlation between histology- and MRI-derived cell sizes and cellularities across all 55 ROIs, with Pearson’s correlation coefficients of 0.61 and 0.78, respectively, both with *p* < 0.0001.

## 4. Discussion

Differences in cell size and cellularity between HCC and other liver lesions or nodules have been reported and used as diagnostic criteria during pathological examination via biopsies [7,8]. However, the lack of a reliable and accurate in vivo method to assess these parameters has hindered the development of non-invasive diagnostic techniques based on cell size or cellularity. Diffusion MRI has been actively investigated for this purpose, as it probes tissue microstructures at a length scale of tens of micrometers. The major limitation of the most used metric—the apparent diffusion coefficient (ADC)—is its lack of specificity in assessing cell size or cellularity, though it is sensitive to the overall effects of these cellular properties [10,33].

Our proposed method, based on diffusion MRI, extracts both cell size and cellularity by fitting diffusion signals acquired at multiple diffusion times and b-values to a biophysical model. This approach overcomes the limitations of conventional diffusion MRI and ADC measurements, providing direct measurements of cell size and cellularity. Our study validates that MR-cytometry-derived cell size and cellularity can serve as non-invasive imaging markers for differentiating HCC from CRN and normal liver tissues, using histology-based simulations and ex vivo experiments with fixed human liver specimens. The next step is to apply MR cytometry in vivo in subjects with HCC and further assess its diagnostic potential, particularly for distinguishing HCC from CRN tissue.

MR cytometry combines diffusion-weighted acquisitions with varying diffusion times, where the range of diffusion times determines the assessable microstructure length scale. For HCC characterization, the relevant microstructure length scale ranges from 5 to 20 µm, corresponding to a diffusion time range of 5 to 70 ms. Clinically, diffusion measurements with relatively long diffusion times (e.g., >10 ms) have been routinely achieved using PGSE sequences. In contrast, measurements with short diffusion times (e.g., <10 ms) historically posed challenges due to the need for high gradient strength and a fast gradient slew rate. However, recent hardware advancements have mitigated this limitation. All major MRI vendors now commonly support gradient strengths up to 80 mT/m and a slew rate ≈ 100 mT/m/s [34,35,36], enabling a diffusion time of ~5 ms with a maximum b-value of ~300 s/mm^2^. The feasibility of MR cytometry has been demonstrated in breast, liver, and prostate studies using clinical 3T scanners, as evidenced by work from our group [15,16,37] and others [21,22,23,24,25].

In HCC characterization, the complex microenvironment of diseased liver tissues—such as cirrhosis, tumors, and iron overload—leads to shorter T2 relaxation times and lower signal-to-noise ratios (SNRs) compared to healthy livers. As our histology-based simulations suggest, an SNR of 20 is necessary to differentiate HCC from CRN tissue and normal liver tissues. Therefore, it is essential to adjust the image resolution and scan time to ensure sufficient SNR for microstructure characterization in patients with cirrhosis or HCC. In cases where a lesion has been identified using high-resolution anatomical imaging (e.g., T1-weighted imaging with or without a contrast agent), a region-of-interest (ROI)-based MR cytometry analysis could be performed to enhance the SNR by the square root of the number of voxels within the ROI.

MR cytometry extracts cellular information from the diffusion time dependency of signal attenuation. Alternative methods to describe this relationship include approximating the diffusion signal attenuation at a single diffusion time as a Gaussian diffusion, which can be described by a mono-exponential decay with the apparent diffusion coefficient (ADC). The ADC spectrum can be expressed as a function of restriction size and diffusion coefficient at infinitely long/short diffusion times, or the ADC spectrum can be characterized by its slope (dispersion rate), which refers to the difference in ADCs divided by the difference in diffusion time. While these methods provide parameters with less clear biophysical meanings, they require fewer data and shorter scan times, potentially lowering the SNR requirements for fitting a more sophisticated model.

A limitation of this study is the lack of investigation into how blood flow and bulk motion influence MR cytometry analysis, which is crucial for in vivo MR cytometry due to the high perfusion and volume fraction of blood in the liver [38]. Our previous work has reported the signal model that includes influences of signal dephasing in the vasculature (or perfusion in tissues) [15]. Briefly, for each diffusion time, the tissue diffusion fraction $\left(1-f_{IVIM}\right)$, where $f_{IVIM}$ represents the blood volume fraction from perfusion and can be approximated by assuming perfusion signals completely diminish when b ≥ 0.2 ms/µm^2^. $f_{IVIM}$ can be estimated by extrapolating the diffusion-weighted signal curves acquired at b-values ≥ 0.2 ms/µm^2^ back to the intercept at b = 0, assuming a mono-exponential decay. Perfusion-free diffusion-weighted signals can then be calculated by dividing the signal curves acquired at b-values ≥ 0.2 ms/µm^2^ by the corresponding ($1-f_{IVIM}$). In addition, the blood volume fraction could assist in diagnosing HCC since HCC is usually hyper-vascularized [39].

The imaging sequences can be further optimized for in vivo MR cytometry of the liver. Since OGSE sequences are flow-compensated, they are less affected by bulk motion and microcirculatory blood flows, while PGSE sequences are not. Therefore, using flow-compensated PGSE sequences would provide more robust diffusion signals [40], improving SNR by minimizing signal decay from microcirculatory blood flows. Additionally, radial imaging techniques (e.g., MultiVane on Philips 3T systems) offer greater immunity against geometric distortions than EPI sequences, oversample the central k-space, and allow for complete data correction between shots, enhancing SNR [41].

In this study, validation through ex vivo MR cytometry results compared to histology confirms the biophysical mechanisms of MR-cytometry-derived parameters. However, determining the exact accuracy of MR-cytometry-derived cell sizes and cellularities remains challenging. Both MRI and histology have inherent biases when measuring average cell size and cellularity in biological tissues. For MRI, the range of cell sizes that can be measured by MR cytometry depends on how far water molecules diffuse, which is determined by the diffusion time and water diffusion coefficient. For the effective diffusion times used in this study (5–70 ms), MR cytometry is sensitive to cell sizes from approximately 5 to 20 µm, with larger cells contributing more to the average MRI-derived cell size due to volume-weighted diffusion signals. The relationship between the average MRI-derived cell size and the true average cell size depends on the distribution of cell sizes. Additionally, water exchange effects for diffusion measurements with longer diffusion times (>10 ms) are included in our signal model, reducing error for slow water exchange but still underestimating the intracellular volume fraction ($v_{in}$) with faster water exchange, leading to underestimations of cellularity. Furthermore, the formula used to calculate cellularity does not account for fat content, resulting in overestimation of cellularity in fatty liver tissues.

For histology, tissue shrinkage during preparation and the fact that tissue sections rarely pass through the center of the cell lead to an underestimation of cell size. Imperfect co-registration between MRI and histology may also contribute to moderate correlation between the two measurements. Despite using a custom tissue sample holder to ensure both MR images and histological sections come from the same location, tissue shrinkage and distortion during preparation complicate registration. The 2D shape registration used in this study matches the outlines of the same specimen in MR and histology images, but errors may still arise when transforming ROIs due to tissue deformation complexities. Moreover, the histological section is thinner than the MR image slice thickness. Given these factors, determining the exact accuracy of MRI-derived parameters is challenging. However, it is more important to assess the repeatability and reproducibility of MR cytometry measurements rather than focusing solely on the exact accuracy of MRI-derived parameters. Our ultimate goal is to distinguish HCC from surrounding liver tissues. Therefore, the key is to ensure that MRI-quantified differences between HCC and other conditions are sufficient to enable accurate classification.

The sample size is a limitation of this study, which precludes a detailed analysis of MR presentations across different HCC differentiation grades or subtypes or other types of liver cancers. In this study, the primary clinical challenge we aim to address is distinguishing early-stage HCC (typically well-differentiated) from cirrhotic regenerative nodules, a critical diagnostic dilemma in HCC surveillance. Effects on characterizing HCC subtypes and other types of liver tumors, such as metastases, cholangiocarcinoma, and hemangioma, using MR cytometry are ongoing.

## 5. Conclusions

This study demonstrates the potential of MR cytometry to differentiate HCC from cirrhotic regenerative nodules by quantifying cell size and cellularity in liver tissues through histology-based simulations and ex vivo experiments. Our findings lay a strong foundation for further research into the role of in vivo MR cytometry in the noninvasive early diagnosis of HCC.

## Data Availability

The de-identified raw data supporting the conclusions of this article will be made available by the authors upon request.

## Abbreviations

- HCC: hepatocellular carcinoma
- IMPULSED: imaging microstructural parameters using limited spectrally edited diffusion
- CRN: cirrhotic regenerative nodule
- ADC: apparent diffusion coefficient
- TDS: temporal diffusion spectroscopy
- OGSE: oscillating gradient spin echo
- PGSE: pulsed gradient spin echo
